# Supplementary material for: Development of Marker Recycling Systems for Sequential Genetic Manipulation in Marine-Derived Fungi Spiromastix sp. SCSIO F190 and Aspergillus sp. SCSIO SX7S7
Source: J Fungi (Basel). 2023 Feb 26;9(3):302. doi: 10.3390/jof9030302 (PMC10059709; doi:10.3390/jof9030302)
Supplement: Supplementary file 1 [file jof-09-00302-s001.zip › jof-2194962-supplementary.pdf]

Table S1 Strains used in this study

Strians	Characteristic(s)	Source
Aspergillus sp. SCSIO SX7S7	Wild-type strain, depsidones producing	Ref [24]
Spiromastix sp. SCSIO F190	Wild-type strain, spiromarmycin producing	Ref [25]
Δ*pyrG-7S7*	Δ*pyrG*::*hph*	This study
7S7-Δ*depD*-Δ*pyrG*	Δ*pyrG*::*hph*; Δ*depD* ::*afpyrG*	This study
7S7-Δ*depH*-Δ*pyrG*	Δ*pyrG*::*hph*; Δ*depH* ::*afpyrG*	This study
7S7-Δ*depH*	Δ*pyrG*::*hph*; Δ*depH*	This study
F190-Δ*spm11*-Δ*amdS*	Δ*spm11*::Δ*amdS*	This study
F190-Δ*spm11*	Δ*spm11*	This study

Table S2 Plasmids used in this study

Plasmids	Characteristic(s)	Source
pBSKII-*toCas9-hph*	pBSKII-PtrPC-Flag-*toCas9*-TtrPC containing the *hph* cassette	Ref [29]
pFC330	sgRNA expression cassette	Ref [30]
pBluescript II SK plasmid	Plasmid for generating *pyrG/amdS* expression cassette	Ref [28]
pBSKII-to*Cas9*-*hph*-sgRNA	Cas9, sgRNA and *hph* expression vector	This study
pBSKII-to*Cas9*-*pyrG*-sgRNA	Cas9, sgRNA and *pyrG* expression vector	This study
pBSKII-to*Cas9*-*amdS*-sgRNA	Cas9, sgRNA and *amdS* expression vector	This study

Table S3. Primers used in this study

Name	Sequence (5' to 3')	Experiment
pks-P*trpc*-F	atatcgaattcctgcagcccgcgcaattaaccctcactaa	Cloning of *pyrG/amdS* expression cassette
pks-P*trpc*-R	ttcgatgcttgggtagaatagg	Cloning of *pyrG/amdS* expression cassette
amdS-F1	cctattctacccaagcatcgaaatgcctcaatcctgggaagaac	Cloning of *amdS* expression cassette
amdS-R1	gctattaaatcactagaaggcactcctatggagtcaccacatttcccagc	Cloning of *amdS* expression cassette
pks-T*trpc*-an-F	gagtgccttctagtgatttaatagctcc	Cloning of *pyrG/amdS* expression cassette
pks-T*trpc*-an-R	ctagaactagtggatcccccggagcattcactaggcaaccat	Cloning of *pyrG/amdS* expression cassette
pyrG-F1	gacttacctattctacccaagcatcgaaatgtcgtccaagtcgcaattgac	Cloning of *pyrG* expression cassette
pyrG-R1	gctattaaatcactagaaggcactctcatgacttgccgcatactctggcc	Cloning of *pyrG* expression cassette
psiI-DR-F	ggccgaaatcggcaaaatcccttatataactaatcaagtttttgggg tcgaggtgccg	Cloning of *pyrG/amdS* expression cassette
DR-R	cctttcgccagctggcgtaa	Cloning of *pyrG/amdS* expression cassette
DR-P*trpc*-F	ttacgccagctggcgaaagggcgcaattaaccctcactaa	Cloning of *pyrG/amdS* expression cassette
psiI-T*trpc*-R	ccctatctcggtctattcttttgatttattataaggagcattcactaggcaaccatgg	Cloning of *pyrG/amdS* expression cassette

7S7-*pyrg*-flag1-R	gacgagcttactcgtttcgtcctcacggactcatcaggtcggCcg gtgatgtctg	Cloning of sgRNA expression cassette
7S7-*pyrg*-flag2-F	acgagtaagctcgtcgtcggcggagacggtgacgtgttttagagc tagaaatagca	Cloning of sgRNA expression cassette
7s7-*pyrg*-test-F	ggctctcgcgcagacgacgc	Amplification of the DNA region surrounding the PAM site of *pyrg-7S7*
7s7-*pyrg*-test-R	gtttcatggggatcccacagtcagct	Amplification of the DNA region surrounding the PAM site of *pyrg-7S7*
7s7-*depD*-crisper-frag1-R	gacgagcttactcgtttcgtcctcacggactcatcagggaaggcg gtgatgtctgctcaa	Cloning of sgRNA expression cassette
7s7-*depD*-crisper-frag2-F	acgagtaagctcgtcggaagggttctgggacctgtgttttagagct agaaatagca	Cloning of sgRNA expression cassette
7s7-*depH*-crisper-frag1-R	gacgagcttactcgtttcgtcctcacggactcatcagggcgggcg gtgatgtctgctcaa	Cloning of sgRNA expression cassette
7s7-*depH*-crisper-frag2-F	acgagtaagctcgtcggcgggatccagggctgctggttttagagc tagaaatagc	Cloning of sgRNA expression cassette
7s7-*depH*-test-F	gctgtcaaacatcctgccca	Amplification of the DNA region surrounding the PAM site of *depH*
7s7-*depH*-test-R	aagcgccactccatctcgat	Amplification of the DNA region surrounding the PAM site of *depH*
7s7-*depD*-test-F	ctcacggtttatctcgtcga	Amplification of the DNA region surrounding the PAM site of *depD*
7s7-*depD*-test-R	aagatgcagcgcaaccagac	Amplification of the DNA region surrounding the PAM site of *depD*
amdS-*pyrG*-DR-test-F	ccaataggccgaaatcggcaaaatccc	Testing the integrity of *pyrG/amdS* expression cassette
amdS-*pyrG*-DR -test-R	gactgggaaaaccctggcgttacccaactta	Testing the integrity of *pyrG/amdS* expression cassette
f190-*spm11*-crisper-frag1-R	gacgagcttactcgtttcgtcctcacggactcatcagggttagcggt gatgtctg	Cloning of sgRNA expression cassette
f190-*spm11*-crisper-frag2-F	acgagtaagctcgtcggttagcatggcgcttgagggttttagagct agaaatagc	Cloning of sgRNA expression cassette
spm11-crisper-test-F	tatgagtcagtgctcacgc	Amplification of the DNA region surrounding the PAM site of *spm11*
spm11-crisper-test- F1	ggttagcatggcgcttgagg	Amplification of the DNA region surrounding the PAM site of *spm11*
spm11-crisper-test-R	cattagcaagctcatccggc	Amplification of the DNA region surrounding the PAM site of *spm11*
ITS1	tccgtaggtgaacctgcgg	Amplification of the ITS region
ITS4	tcctccgcttattgatatgc	Amplification of the ITS region

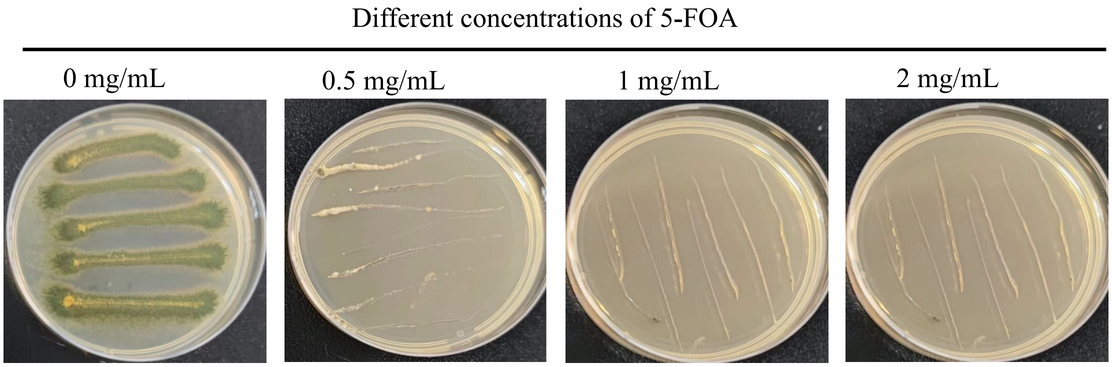

Figure S1. Growth of *Aspergillus* sp. SCSIO SX7S7 on PDA plate with different concentrations of 5-FOA. The spores of *Aspergillus* sp. SCSIO SX7S7 were streaked on the PDA plate with different concentrations of 5-FOA and incubated at 28°C for 7 days.

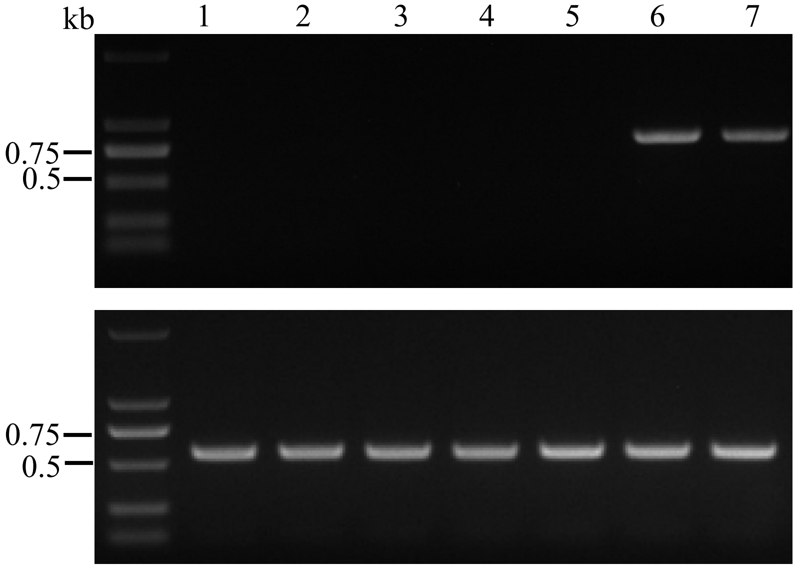

Figure S2. Verification of the Δ*depH* mutants by genotyping PCR. Upper panel: DNA amplification of the DNA region surrounding sgRNA binding site of the six clones picked out from the regeneration plate using primers flanking the PAM site in *depH* gene; Lower panel: Checking the DNA template quality of the tested samples using primers flanking the ITS region. PCR amplification with the same primers of DNA from the wild type strain served as positive controls. (Lane 1–6: tested transformants; lane 7: wild-type strain.)

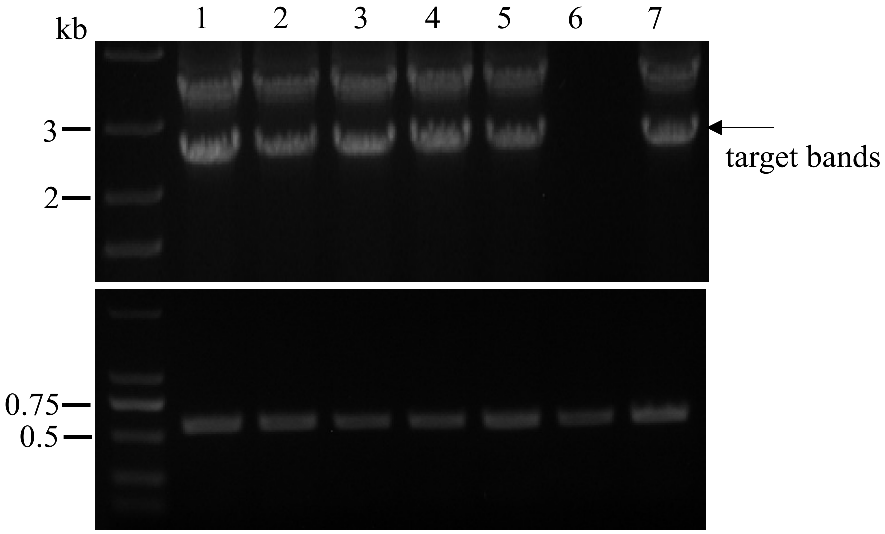

Figure S3. Verification of the integrity of DR flanked *AfpyrG* expression cassette. Upper panel: PCR was carried out using the primers target the complete DRs franked *AfpyrG* marker expression cassette. Lower panel: the DNA template quality of tested samples was validated using primers flanking the ITS region. The wild type strain was set as negative control and plasmid containing DRs franked *AfpyrG* marker was set as positive control. (Lane 1-5: five independent Δ*depH* mutants; Lane 6: negative control； Lane 7: positive control.)

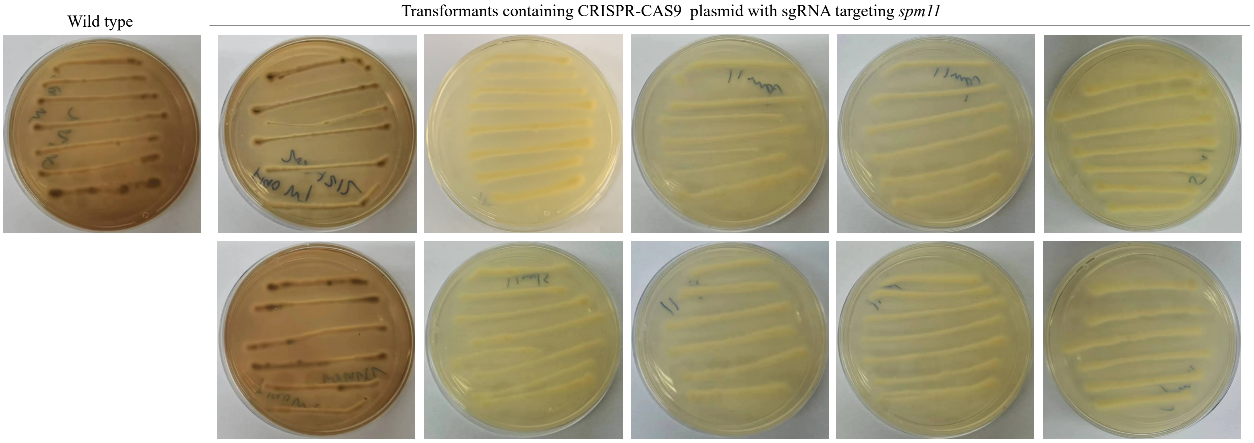

Figure S4. Growth of *Spiromastix* sp. SCSIO F190 strains. The wild type strain and transformants containing CRISPR-CAS9 plasmid with sgRNA targeting *spm11* were grown on PDA for 10 days.

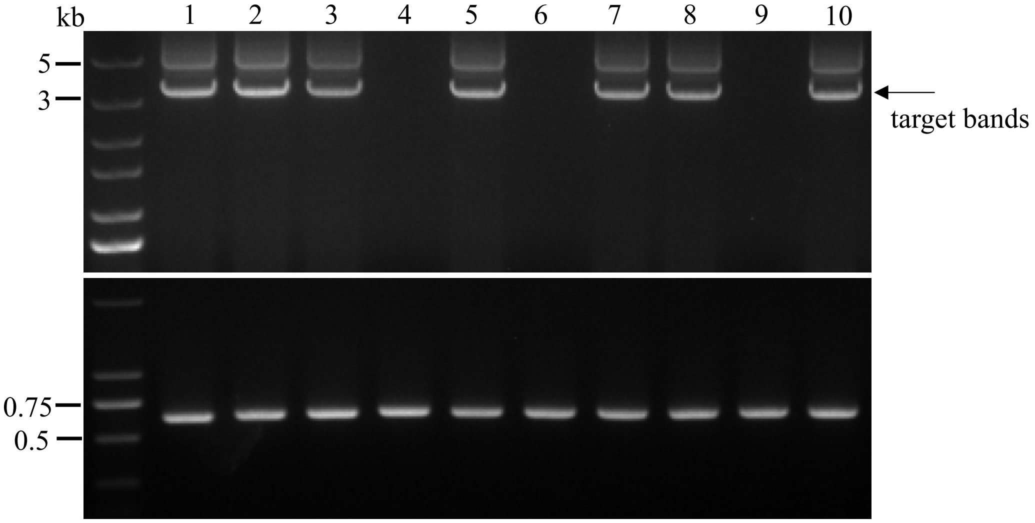

Figure S5. Verification of integrity of DR flanked *AnamdS* expression cassette. Upper panel: PCR was carried out using the primers target the complete DRs franked *AnamdS* marker expression cassette. Lower panel: DNA template quality of tested samples was validated using primers flanking the ITS region. The wild type strain was set as negative control and plasmid containing DRs franked *AnamdS* marker was set as positive control. (Lane 1-8: eight independent Δ*spm11* mutants; Lane 9: negative control；Lane 10: positive control.)
